# Impact of extended equilibration periods on in vitro post-thaw sperm quality in rams

**DOI:** 10.1007/s11259-026-11327-9

**Published:** 2026-06-24

**Authors:** Firat Korkmaz, Sukru Gungor, Muhammed Enes Inanc, Mine Herdogan, Hasan Ali Cay, Feyzanur Mart, Durmus Kahraman, Ufuk Kaya

**Affiliations:** 1 Department of Reproduction and Artificial Insemination, Faculty of Veterinary Medicine, Burdur Mehmet Akif Ersoy University, TR-15030 Burdur , Türkiye; 2 Department of Statistics, Faculty of Veterinary Medicine, Hatay Mustafa Kemal University, TR-31300 Hatay, Türkiye

**Keywords:** Equilibration durations, Flow cytometry, Freezing, Ram semen

## Abstract

Equilibration, a critical phase in cryopreservation, allows sperm cells to adapt to cryoprotective agents (CPAs) before freezing. This study investigated the impact of extended equilibration durations on the quality of frozen-thawed ram spermatozoa. Semen samples from five healthy Merino rams were pooled, extended with a Tris-based extender, and equilibrated for 3 h (control), 24, 48, or 72 h. post-equilibration and post-thaw sperm quality parameters, including motility, plasma membrane and acrosome integrity (PMAI), high mitochondrial membrane potential (HMMP), mitochondrial reactive oxygen species (MROS) levels, lipid peroxidation (LPO), and intracellular calcium levels (ICL) were analysed. Short equilibration durations (3–24 h) preserved motility, PMAI, and HMMP while minimizing MROS production, lipid peroxidation, and calcium influx. Conversely, prolonged equilibration (48–72 h) leads to CPA toxicity, oxidative stress, and premature capacitation-like changes, impairing sperm viability. Notably, 3-hour equilibration maintained post-thawed sperm with the highest motility (83.96 ± 1.87%) and lowest lipid peroxidation levels (11.46 ± 2.12%). These findings emphasize that shorter equilibration times optimize CPA penetration and minimize cryodamage, thereby enhancing post-thaw sperm function. These results provide valuable insights into refining cryopreservation protocols to improve freezing -thawing outcomes in ram sperm freezing centres.

**Supplementary Information:**

The online version contains supplementary material available at https://doi.org/10.1007/s11259-026-11327-9.

## Introduction

Cryopreservation of ram semen is essential in modern animal breeding and genetic management programs (Szymanowicz et al. 2022). It allows long-term preservation and global distribution of valuable genetic material, supporting breeding efforts across time and geography (Ahmad et al. 2015). Among the stages of cryopreservation, equilibration—the period when spermatozoa are exposed to cryoprotective agents (CPAs) at cooling temperatures before freezing—plays a decisive role in determining post-thaw sperm quality (Paul et al. 2020).

During equilibration, sperm cells adapt to the osmotic and biochemical effects of CPAs such as glycerol, which protect against ice crystal formation and osmotic stress (Medeiros et al. 2002). This phase allows CPA penetration and the redistribution of intracellular water and solutes, stabilizing membranes before freezing. Insufficient equilibration can cause incomplete CPA permeation and osmotic injury, while prolonged exposure induces CPA toxicity, oxidative stress, and premature capacitation-like changes that impair sperm function (Leahy and Gadella 2011; Yeste 2016).

Equilibration also permits physiological and biochemical adjustments that enhance tolerance to cooling and thawing (Medeiros et al. 2002; Lee et al. 2019). Cellular dehydration during this stage reduces intracellular ice formation and preserves structural integrity. However, the optimal equilibration time varies with species, extender composition, and CPA concentration. In rams, reported equilibration durations typically range between 2 and 4 h (Ashrafi et al. 2011; Bucak et al. 2013; Joshi et al. 2008), though some studies describe benefits with up to 24 h (Câmara et al. 2011; Paul et al. 2020).

Research on other species, particularly bulls, shows that extending equilibration may improve post-thaw motility and fertility by promoting gradual adaptation to CPAs. Murphy et al. (2018) found that longer equilibration enhanced sperm function and conception rates following artificial insemination. Nevertheless, ram sperm are less cryotolerant than those of many species (Szymanowicz et al. 2022), so extended equilibration might increase vulnerability to CPA-induced stress.

Optimizing equilibration duration is thus critical for achieving maximal cryosurvival. The challenge lies in balancing adequate CPA penetration with the prevention of oxidative and metabolic stress. Although prior research has clarified some effects of equilibration time on membrane and motility parameters, its impact on mitochondrial function, ROS generation, and calcium homeostasis remains poorly understood. In this context, extended equilibration durations, although not routinely applied in practice, may serve as a useful experimental model to better understand the progressive cellular responses to prolonged CPA exposure. Therefore, the present study aimed to provide a comprehensive, multiparametric evaluation of plasma membrane and acrosome integrity, mitochondrial function, oxidative stress, and calcium dynamics by assessing the influence of extended equilibration durations (3, 24, 48, and 72 h) on post-thaw ram sperm quality.

## Materials and methods

The study was conducted using five adult and healthy Merino rams housed on a private farm in Burdur province. Rams, aged between two and five years, were selected from animals that exhibited no health or fertility problems.

### Semen collection and evaluation 

Ejaculates were collected using an electroejaculator (MiniTube, Ref: 11900/0000, Germany) once a week during the breeding season, yielding a total of five ejaculates from each of the five rams. After semen collection, all samples were stored at 37 °C and transported to the laboratory within 30 min for semen sample quality assessment, including assessment of volume (0.5 to 1.5 mL), concentration (≥ 1 × 10^9^ spermatozoa/mL), and total motility (≥ 80%). Normozoospermic ejaculates (defined as samples meeting minimum criteria for volume, concentration, and motility) were pooled and analysed. The pooled ejaculates that were separated into four aliquots and extended using a Tris-based extender with 15% egg yolk and 6% glycerol (Avdatek et al. 2023).

The diluted ejaculates were gradually cooled and stored at 4 °C during the equilibration period (3, 24, 48, and 72 h). At the end of each equilibration period, half of the straws were used for direct analysis, while the remaining half were frozen. For freezing, the straws were placed horizontally on a rack approximately 5 cm above the surface of liquid nitrogen (− 196 °C), exposed to liquid nitrogen vapor (approximately − 120 °C) for 12 min, and then plunged into liquid nitrogen for storage. Frozen straws were thawed in a water bath at 37 ± 2 °C for 25–30 s prior to evaluation (Inanc et al. 2019). Spermatological quality parameters were analysed at the end of equilibration and after thawing.

### Motility

Sperm motility was assessed subjectively by examining at least five microscopic fields of the semen sample under 400x magnifications of a phase-contrast microscope with a heating plate at 37 °C, and the mean of the motility values in the fields was recorded as the subjective motility ratio (Inanc et al. 2019).

### Flow cytometric analyses

**Fig. 1:**
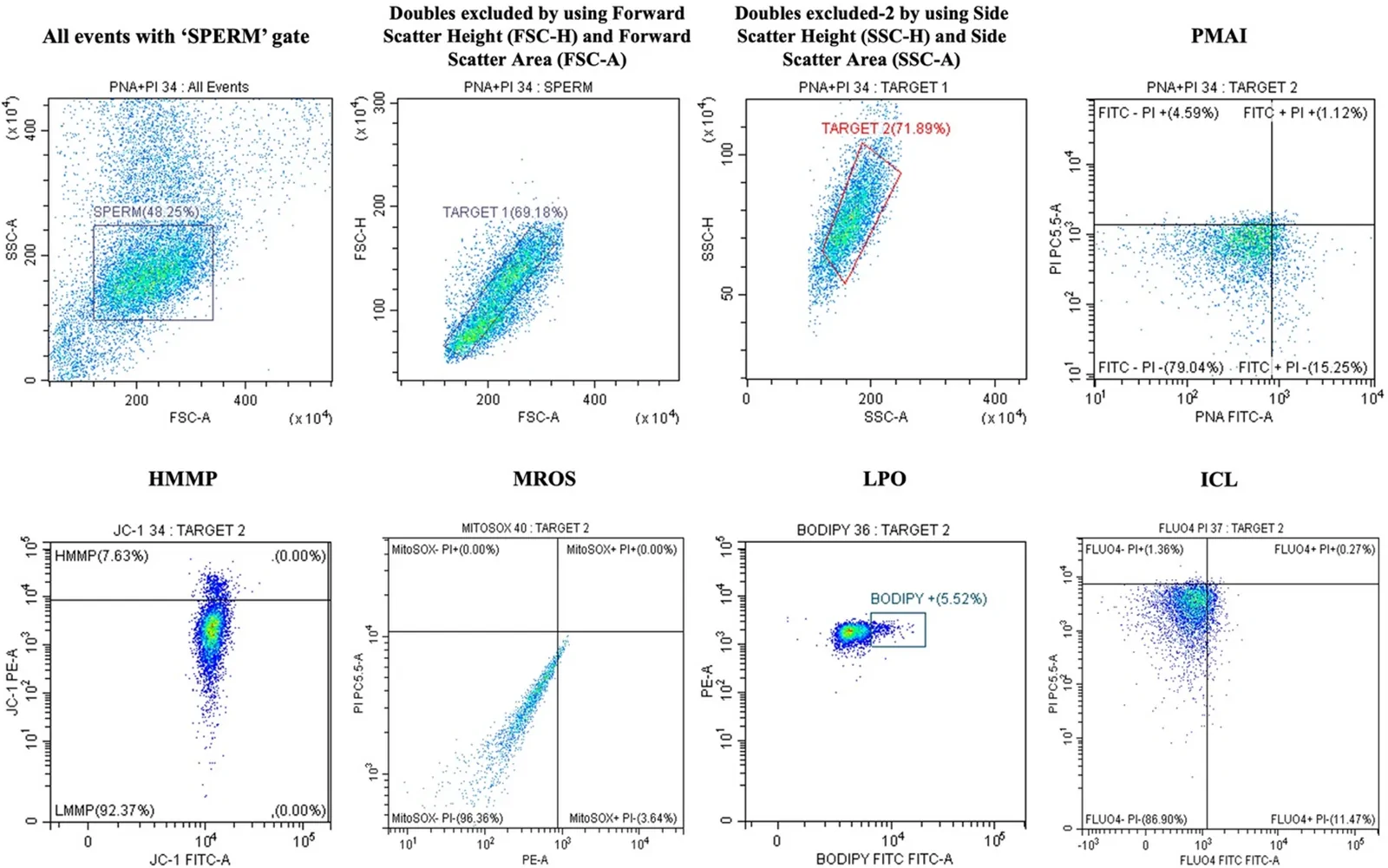
Representative flow cytometry dot plots used for the assessment of sperm plasma membrane and acrosome integrity (PMAI), high mitochondrial membrane potential (HMMP), mitochondrial reactive oxygen species (MROS), lipid peroxidation (LPO), and intracellular calcium levels (ICL) in the experimental groups

Analyses were conducted using a CytoFLEX flow cytometer (Beckman Coulter, CA, United States) that featured emission filters at 610 ± 20 nm, 585 ± 42 nm, 525 ± 40 nm, and a 50 mW (488 nm) argon laser output. Each analysis involved examining an average of 10,000 spermatozoa. Pseudo-color plots were employed to compare the side scatter area (SSC-A) with the forward scatter area (FSC-A) of the sperm cells, aiding in the selection process. To eliminate doublets from the analyses, the forward scatter height (FSC-H) and forward scatter area (FSC-A) were utilized. All results were obtained using the CytExpert 3.1 software. Prior to analysis, all samples were incubated at 37 °C for 15 min in a dark room (Bucher et al. 2019; Korkmaz et al. 2017) (Fig. 1).

### Sperm plasma membrane and acrosome integrity assessment

Plasma membrane and acrosome integrity (PMAI) of sperm was assessed using FITC-PNA (Sigma, L7381; final concentration: 100 µg/mL) in combination with propidium iodide (PI, L7011 Molecular Probes, Invitrogen; final concentration: ~18 µM) fluorescence staining. Briefly, samples were diluted to 5 × 10⁶ spermatozoa/mL in 492 µL PBS and mixed with 5 µL FITC-PNA and 3 µL PI.

### High mitochondrial membrane potential

Sperm high mitochondrial membrane potential (HMMP) was determined using 5,5′,6,6′-tetrachloro-1,1′,3,3′-tetramethylbenzimidazolyl carbocyanine iodide (JC-1) (Invitrogen, T3198 Molecular Probes; final concentration: ~10 µM). Samples were diluted to 5 × 10⁶ spermatozoa/mL in 492 µL PBS and mixed with 5 µL JC-1.

### Mitochondrial reactive oxygen species analyses

Mitochondrial reactive oxygen species (MROS) levels in ram spermatozoa were assessed using MitoSOX Red mitochondrial superoxide indicator (M36008, Molecular Probes, Invitrogen; final concentration: ~50 nM) in combination with propidium iodide (PI; final concentration: ~18 µM). Semen samples were diluted to 5 × 10⁶ spermatozoa/mL in 492 µL PBS, and 5 µL MitoSOX Red and 3 µL PI were added.

### Lipid peroxidation levels

Lipid peroxidation (LPO) was assessed using Bodipy and SYBR-14 staining, following a previously described method with slight modifications (Korkmaz et al. 2017). Samples were diluted to 5 × 10⁶ spermatozoa/mL in 492 µL PBS, and 5 µL Bodipy (final concentration: ~10 µM) and 5 µL SYBR-14 (final dilution: 1:10) were added.

### Spermatozoa Ca^*+ 2*^ levels

Intracellular Ca²⁺ (ICL) levels in spermatozoa were determined using Fluo-4 (Invitrogen, F14201, Molecular Probes; final concentration: ~10 µM) in combination with propidium iodide (PI, L7011 Molecular Probes, Invitrogen; final concentration: ~18 µM) fluorescence staining. Briefly, samples were diluted to 5 × 10⁶ spermatozoa/mL in 492 µL PBS and mixed with 5 µL Fluo-4 and 3 µL PI.

## Statistical analyses

Data were analysed using IBM SPSS Statistics for Windows, version 23.0. Statistical significance was set at *p* < 0.05. All values are presented as mean ± standard error of the mean. The overall effect of group (equilibration and frozen-thawed), time, and their interaction term on the change in motility, PMAI, HMMP, MROS, LPO, and ICL between 3, 24, 48, and 72 h were analysed using a mixed-effect linear model, with ram as a random effect and group, time, and the interaction term as fixed effects. Variance components were used as the covariance structure in the established model because they resulted in the lowest Akaike Information Criterion (AIC). Pairwise comparisons were assessed using Bonferroni adjustment simple effect analysis.

## Results

### Motility

**Fig. 2:**
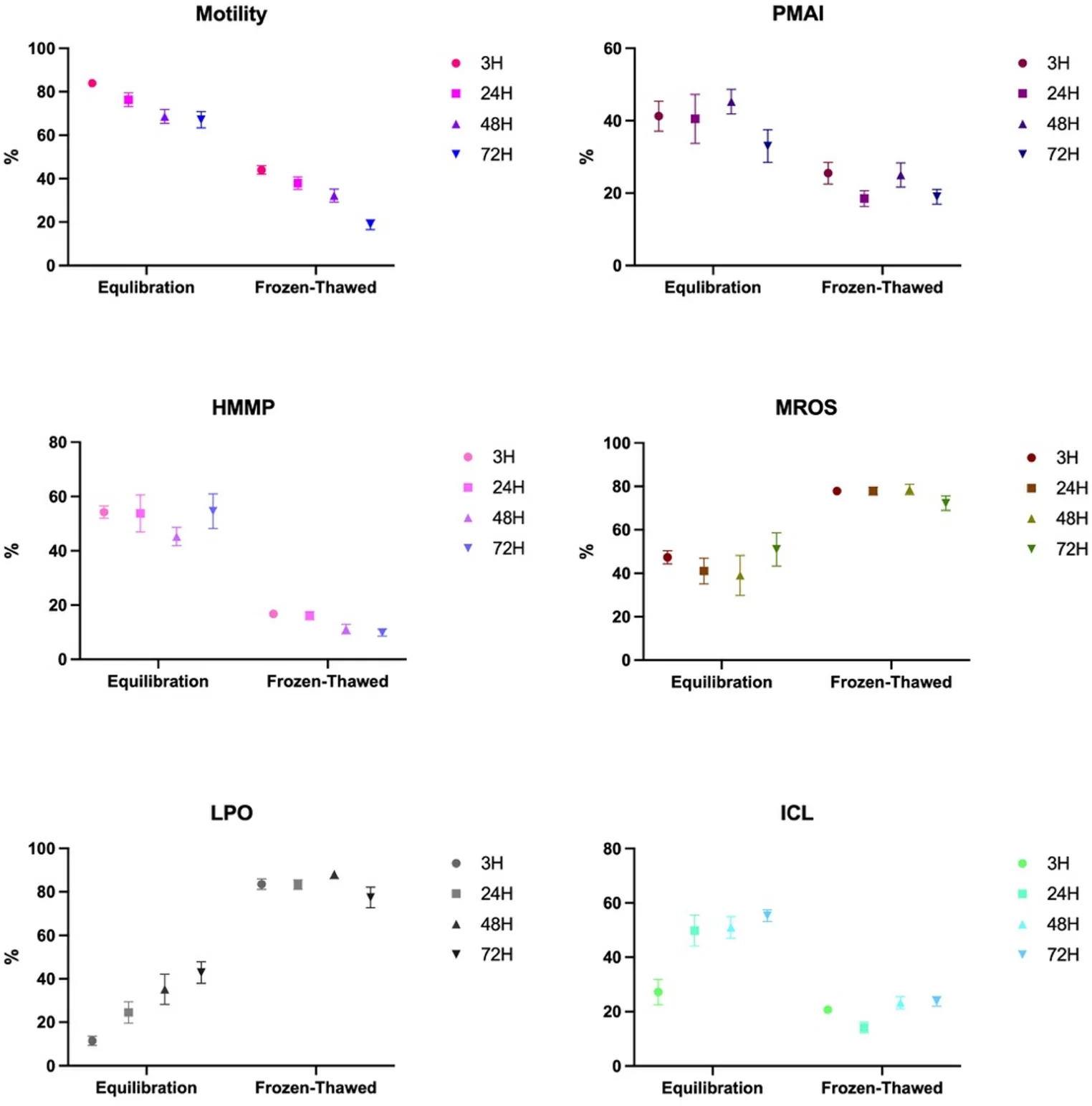
Effects of equilibration time (3, 24, 48, and 72 h) on sperm quality parameters. Panels show motility (%), plasma membrane and acrosome integrity (PMAI, %), high mitochondrial membrane potential (HMMP, %), mitochondrial reactive oxygen species (MROS, %), lipid peroxidation (LPO, %), and intracellular calcium levels (ICL, %). Data are presented as mean ± SEM PMAI: Plasma membrane and acrosome intact sperm, HMMP: High mitochondrial membrane potential, MROS: Mitochondrial reactive oxygen species, LPO: Lipid peroxidation, ICL: Intracellular calcium levels

When the post- equilibration results were analysed, the 3 h equilibration (83,96 ± 1,87%) showed higher motility than the 48 h (68,64 ± 3,22%) and 72 h (67,14 ± 3,76%) (*P* < 0.001). Although 3 h showed higher motility results than the 24 h (76,32 ± 3,13%), no statistical difference was observed between the two equilibration times (*P* = 0.115). In addition, the 24 h showed higher motility results than the 72 h equilibration (*P* < 0.001). Post-thaw motility results were similar to those obtained after equilibration. Post-thaw motility results were 43.97 ± 1.95%, 37.96 ± 2.87%, 32.24 ± 2.96%, 18.76 ± 2.19%, respectively (Table 1; Fig. 2).

**Table 1:** Impact of Extended Equilibration Duration on Post-Thaw Sperm Quality Parameters

Parameters (%)	Group	Time				LS Mean (Group)	p		
		3 h	24 h	48 h	72 h		Group	Time	G x T
Motility	Post-Equilibration	83,96 ± 1,87 ^a,A^	76,32 ± 3,13 ^ab,A^	68,64 ± 3,22 ^bc, A^	67,14 ± 3,76 ^c,A^	74,01 ± 2,09	<0,001	<0,001	0,161
	Post-thaw	43,97 ± 1,95 ^a,B^	37,96 ± 2,87 ^ab,B^	32,24 ± 2,96 ^bc,B^	18,76 ± 2,19 ^c,B^	32,86 ± 1,91			
LS Mean (Time)		57,30 ± 5,23	50,75 ± 5,28	43,61 ± 4,88	33,88 ± 6,07				
PMAI	Post-Equilibration	41,25 ± 4,15 ^A^	40,50 ± 6,70 ^A^	45,25 ± 3,35 ^A^	33,00 ± 4,51 ^A^	40,47 ± 2,45	<0,001	0,073	0,661
	Post-thaw	25,50 ±2,99^B^	18,50 ± 2,19 ^B^	25,00 ± 3,32 ^B^	19,00 ± 2,03 ^B^	22,23 ± 1,44			
LS Mean (Time)		30,00 ± 3,07	24,79 ± 3,60	30,40 ± 3,48	22,82 ± 2,66				
HMMP	Post-Equilibration	54,25 ± 2,29 ^A^	53,75 ± 6,85 ^A^	45,25 ± 9,52 ^A^	54,60 ± 6,35 ^A^	52,12 ± 3,19	<0,001	0,103	0,367
	Post-thaw	16,78 ± 1,38 ^B^	16,11 ± 1,49 ^B^	11,18 ± 1,76 ^B^	9,90 ± 1,28 ^B^	13,28 ± 0,87			
LS Mean (Time)		28,31 ± 5,12	27,69 ± 5,46	20,27 ± 4,80	24,80 ± 6,02				
MROS	Post-Equilibration	47,40 ± 3,01 ^B^	41,00 ± 5,95 ^B^	39,00 ± 9,17 ^B^	51,00 ± 7,65 ^B^	44,88 ± 3,00	<0,001	0,708	0,09
	Post-thaw	77,89 ± 2,43 ^A^	77,89 ± 1,84 ^A^	78,56 ± 2,38 ^A^	72,27 ± 3,38 ^A^	76,42 ± 1,36			
LS Mean (Time)		67,00 ± 4,45	64,71 ± 5,41	68,67 ± 5,79	66,60 ± 3,96				
LPO	Post-Equilibration	11,46 ± 2,12 ^c, B^	24,50 ± 4,93 ^bc, B^	35,19 ± 6,93 ^ab, B^	42,87 ± 4,93 ^a, B^	29,67 ± 3,73	<0,001	≤0,001	<0,001
	Post-thaw	83,56 ± 2,45 ^A^	83,39 ± 2,25 ^A^	88,17 ± 1,93 ^A^	77,41 ± 4,72 ^A^	83,40 ± 1,52			
LS Mean (Time)		62,96 ± 9,21	66,56 ± 7,65	71,62 ± 6,78	65,08 ± 5,71				
ICL	Post-Equilibration	27,25 ± 4,68 ^b^	49,80 ± 5,72 ^a, A^	51,00 ± 3,99 ^a, A^	55,33 ± 2,19 ^a, A^	45,82 ± 3,39	<0,001	<0,001	<0,001
	Post-thaw	20,70 ± 1,56 ^ab^	14,13 ± 1,95 ^b, B^	23,33 ± 2,22 ^a, B^	23,80 ± 1,78 ^a, B^	20,76 ± 1,09			
LS Mean (Time)		22,57 ± 1,82	27,85 ± 5,54	33,21 ± 4,15	31,08 ± 4,09				

Different lowercase letters (a, b, c) within the same row indicate significant differences between equilibration times (P < 0.05). A, B, C – uppercases in the same row represent the difference between groups in each time period (P < 0.05). *PMAI* Plasma membrane and acrosome intact sperm, *HMMP* High mitochondrial membrane potential, *MROS* Mitochondrial reactive oxygen species, *LPO* Lipid peroxidation, *ICL* Intracellular calcium levels

### Sperm plasma membrane and acrosome integrity

When the post-equilibration PMAI results were examined, no statistical difference was found between the time points (*P* = 0.073). The PMAI values of the 3 h, 24 h, 48 h and 72 h were 41,25 ± 4,15%, 40,50 ± 6,70%, 45,25 ± 3,35%, 33,00 ± 4,51%, respectively. Similarly, there was no statistical difference between the times in the post-thaw PMAI results, which were 25.50 ± 2.99%, 18.50 ± 2.19%, 25.00 ± 3.32%, and 19.00 ± 2.03%, respectively (*P* = 0.073) (Table 1; Fig. 2).

### High mitochondrial membrane potential

The HMMP values also displayed significant variations among equilibration times (*P* < 0.001). The HMMP values were relatively stable across 3 h (54.25 ± 2.29%), 24 h (53.75 ± 6.85%), and 72 h (52.12 ± 3.19%), but showed a slight decline at 48 h (45.25 ± 9.52%) after equilibration. However, there was no statistically significant difference between the time points (*P* = 0.103).

The post-thaw values significantly decreased across all equilibration durations. The 3 h retained the highest HMMP (16.78 ± 1.38%), followed by 24 h (16.11 ± 1.49%). A notable decline occurred at 48 h (11.18 ± 1.76%) and 72 h (9.90 ± 1.28%). However, no time effect was observed (*P* = 0.103). Notably, HMMP values at 48 h during the post-equilibration period exhibited greater variability, as indicated by wider SEM values (Table 1; Fig. 2).

### Mitochondrial ROS production

When MROS levels were analysed, no difference was found between the time during both post-equilibration and post-thaw periods (*P* = 0.708). During post-equilibration, MROS levels were 47.40 ± 3.01%, 41.00 ± 5.95%, 39.00 ± 9.17%, 51.00 ± 7.65%, and during post-thaw 77.89 ± 2.43%, 77.89 ± 1.84%, 78.56 ± 2.38%, 72.27 ± 3.38%, respectively. The lowest MROS value at post-equilibration was at the 48 h, while it was in the 72 h at post-thaw (Table 1; Fig. 2).

### Lipid peroxidation levels

When LPO levels were analysed, the 3 h (11.46 ± 2.12%) had lower levels compared to the 48 h (35.19 ± 6.93%) and 72 h (42.87 ± 4.93%) at post-equilibration. The 24 h (24.50 ± 4.93%) also had lower LP levels than the 72 h (*P* ≤ 0.001). However, by post-thaw, the LPO levels of the equilibration times were 83.56 ± 2.45%, 83.39 ± 2.25%, 88.17 ± 1.93%, 77.41 ± 4.72%, respectively. Although there was no statistically significant difference between the time points, the lowest LP level was observed at the 72 h (*P* = 0.571) (Table 1; Fig. 2).

### Intracellular calcium levels

When ICL were analysed, 3 h (27.25 ± 4.68%) gave the lowest results for post-equilibration compared to the other times (*P* < 0.001), while the results for 24 h (49.80 ± 5.72%), 48 h (51.00 ± 3.99%) and 72 h (55.33 ± 2.19%) were similar. However, these results differed after thawing. The 24 h (14.13 ± 1.95%) showed lower ICL levels compared to the 48 h (23.33 ± 2.22%) and 72 h (23.80 ± 1.78%) (*p* < 0.001). Although ICL levels were lower at 24 h compared than at 3 h (20.70 ± 1.56%), this difference was not significant (*P* = 0.239) (Table 1; Fig. 2).

## Discussion

Equilibration time is a major determinant of cryopreservation efficiency and profoundly influences post-thaw sperm quality (Pieper et al. 2023). Its optimal duration depends on the type and concentration of cryoprotective agents (CPAs), temperature, and sperm membrane composition (Olğaç et al. 2017; Purdy et al. 2010). In this study, sperm motility decreased significantly with equilibration times longer than 24 h, whereas plasma membrane and acrosome integrity (PMAI) remained statistically unaffected. Although not significant, mitochondrial ROS (MROS) and lipid peroxidation (LPO) levels were slightly lower at 72 h, and intracellular calcium (ICL) was reduced at 24 h compared with 3 h.

Prolonged equilibration appeared less favorable for maintaining motility, consistent with previous reports identifying 2–4 h as the optimal range for rams (Ashrafi et al. 2011; Bucak et al. 2013). Câmara et al. (2011) reported no significant differences in motility among 0, 12, and 24 h equilibration periods, whereas Paul et al. (2020) found superior post-thaw motility at 22 h. Conversely, Vozaf et al. (2021) observed improved motility after 6 h. Such discrepancies likely reflect differences in semen extender composition, CPA concentration, and equilibration temperature, all of which can markedly influence cryosurvival outcomes.

The observed motility decline after 24 h may reflect cumulative CPA toxicity and oxidative stress (Leahy and Gadella 2011; Yeste 2016). Although MROS and LPO differences were not statistically significant, prolonged CPA exposure is known to destabilize plasma and mitochondrial membranes, leading to decreased ATP production and impaired flagellar motion (Davis Volk and Moreland 2014; Joshi et al. 2008). Extended equilibration may also prolong the contact between spermatozoa and ROS-generating CPAs, further enhancing lipid peroxidation and metabolic exhaustion. These combined effects likely compromise the energy-dependent mechanisms that sustain motility, explaining the reduced cryosurvival observed after extended equilibration.

Despite the evident reduction in motility at longer equilibration durations, plasma membrane and acrosome integrity (PMAI) remained statistically unchanged across all equilibration times. Both post-equilibration and post-thaw PMAI values showed no significant differences (*P* > 0.05), suggesting that prolonged exposure to cryoprotectants primarily affects motility-related metabolic pathways rather than gross membrane damage. This apparent stability in PMAI may reflect a transient compensatory response of the sperm membrane, maintaining structural integrity despite increasing metabolic and oxidative stress.

In bulls, motility and PMAI have been reported to improve with equilibration periods up to 96 h (Fleisch et al. 2017); however, this trend is not reproduced in rams, likely due to species-specific differences in membrane lipid architecture. Ram spermatozoa contain a higher proportion of polyunsaturated fatty acids, which confer greater membrane fluidity but simultaneously render the membrane more susceptible to peroxidation and osmotic stress (Mandal et al. 2014; Murawski et al. 2015). Such intrinsic membrane characteristics, together with limited antioxidant defenses, may account for the observed preservation of PMAI values despite a decline in motility. Moreover, proteomic remodeling during equilibration, as demonstrated by Peris-Frau et al. (2019), may influence the molecular composition of the membrane and cytoskeleton, altering sperm functionality after thawing without producing overt structural damage.

The gradual increase in lipid peroxidation (LPO) observed during equilibration, particularly at 48 h and 72 h, indicates that oxidative processes intensify with prolonged CPA exposure. The markedly higher LPO levels at these later stages suggest early onset of oxidative stress even before freezing. Such elevation likely reflects the cumulative generation of reactive oxygen species (ROS) during the equilibration phase, which can initiate peroxidative chain reactions targeting the sperm’s unsaturated membrane lipids. Ram spermatozoa are especially vulnerable to this process because their plasma membranes contain a high proportion of polyunsaturated fatty acids, which confer flexibility but markedly increase oxidative susceptibility (Talevi et al. 2013).

Interestingly, post-thaw LPO values did not differ significantly among groups, although the 72 h sample exhibited the lowest mean level. Although post-thaw LPO did not differ significantly among groups, the numerically lower value at 72 h may reflect the loss of severely damaged cells, but this hypothesis requires direct testing. Overall, these findings suggest that while longer equilibration promotes early oxidative membrane damage, subsequent freezing may mask this effect by eliminating the most severely compromised spermatozoa. Thus, the balance between adequate CPA permeation and minimization of oxidative stress is critical for preserving sperm function.

Intracellular calcium levels (ICL) increased progressively with longer equilibration, reaching their highest values at 72 h. During equilibration, sperm held for 3 h exhibited the lowest calcium levels, whereas 24 h, 48 h, and 72 h groups showed comparably elevated concentrations (*P* < 0.001). After thawing, this pattern partially shifted: sperm equilibrated for 24 h maintained significantly lower ICL than those held for 48–72 h (*P* < 0.001), suggesting that the 24 h period better preserves calcium regulation during cryopreservation.

The progressive calcium accumulation observed with extended equilibration likely reflects increased membrane permeability and disturbed ion channel activity caused by prolonged CPA exposure. Elevated intracellular calcium can trigger premature capacitation-like changes, destabilize plasma membranes, and deplete ATP reserves required for motility and fertilization (Leahy and Gadella 2011). Conversely, maintaining moderate calcium levels, as observed at 24 h, may support optimal metabolic activity and mitochondrial function, enhancing post-thaw sperm performance. These findings underscore the importance of controlling equilibration duration to prevent calcium overload and ensure proper homeostasis throughout the cryopreservation process.

In conclusion, the present study demonstrates that equilibration time is a critical determinant of cryopreservation efficiency in ram sperm, exerting distinct effects on motility, oxidative status, mitochondrial function, and calcium homeostasis. Equilibration periods between 3 and 24 h provided the most favorable balance, preserving sperm functionality while limiting oxidative and metabolic stress. In contrast, prolonged equilibration (48–72 h) induced increased cellular stress and reduced post-thaw motility without markedly affecting plasma membrane and acrosome integrity. These findings suggest that limiting equilibration to within 24 h is optimal for maintaining sperm functional competence and ensuring effective CPA adaptation. However, the use of subjective motility assessment and the relatively small sample size with pooled ejaculates may limit the generalizability of the findings. Further studies incorporating objective motility analysis (CASA), molecular approaches, and artificial insemination trials are warranted to confirm in vivo relevance.

## Supplementary Information

Below is the link to the electronic supplementary material.

Supplementary Material 1 (DOCX 16.3 KB)

## Data Availability

No datasets were generated or analysed during the current study.
