# Supplementary material for: Impact of extended equilibration periods on in vitro post-thaw sperm quality in rams
Source: Vet Res Commun. 2026 Jun 24;50(5):418. doi: 10.1007/s11259-026-11327-9 (PMC13294172; doi:10.1007/s11259-026-11327-9)
Supplement: Supplementary file 1 — Supplementary Material 1 (DOCX 16.3 KB) [file 11259_2026_11327_MOESM1_ESM.docx]

**Impact of extended equilibration periods on post-thaw sperm quality in rams**

**Fırat Korkmaz^1a^*, Şükrü Güngör^1b^, M. Enes Inanç^1c^, Mine Herdoğan^1d^, Hasan Ali Çay^1e^, Feyzanur Mart^1f^, Durmus Kahraman^1g^, Ufuk Kaya^2h^**

^1^ Burdur Mehmet Akif Ersoy University, Faculty of Veterinary Medicine, Department of Reproduction and Artificial Insemination, Burdur, Turkiye

^2^ Hatay Mustafa Kemal University, Faculty of Veterinary Medicine, Department of Statistics, Hatay, Turkiye

^a^ ORCID: 0000-0002-9600-908X; ^b^ ORCID: 0000-0003-3460-522X; ^c^ ORCID: 0000-0001-6954-6309, ^d^ ORCID: 0000-0003-0911-3901; ^e^ ORCID: 0000-0003-1622-2719; ^f^ ORCID: 0000-0002-9788-3238, ^g^ ORCID: 0000-0003-1739-9014; ^h^ ORCID: 0000-0002-4805-0993

*Corresponding author: [fkorkmaz@mehmetakif.edu.tr](mailto:fkorkmaz@mehmetakif.edu.tr)
